# Transforming youth mental health care in a semi‐urban and rural region of Canada: A service description of ACCESS Open Minds Chatham‐Kent

**DOI:** 10.1111/eip.12818

**Published:** 2019-06-27

**Authors:** Paula Reaume‐Zimmer, Ranjith Chandrasena, Ashok Malla, Ridha Joober, Patricia Boksa, Jai L. Shah, Srividya N. Iyer, Shalini Lal

**Affiliations:** ^1^ Mental Health and Addiction Services Bluewater Health and Canadian Mental Health Association Lambton‐Kent Ontario Canada; ^2^ ACCESS Open Minds (Pan‐Canadian Youth Mental Health Services Research Network) Douglas Mental Health University Institute Montreal Quebec Canada; ^3^ Shulich School of Medicine Western University London Ontario Canada; ^4^ Department of Psychiatry McGill University Montreal Quebec Canada; ^5^ Prevention and Early Intervention Program for Psychosis (PEPP) Douglas Mental Health University Institute Montreal Quebec Canada; ^6^ School of Rehabilitation, Faculty of Medicine University of Montreal Montreal Quebec Canada; ^7^ Centre de Recherche du Centre Hospitalier de l'Université de Montréal (CRCHUM) Montreal Quebec Canada

**Keywords:** access, community participation, health care quality, mental health services, youth mental health, Canada

## Abstract

**Aim:**

This study describes how mental health services for youth are being transformed within the context of a semi‐urban and rural region of Canada (Chatham‐Kent, Ontario), based on the framework of ACCESS Open Minds (ACCESS OM), a pan‐Canadian youth mental health research and evaluation network.

**Methods:**

Transformation has focused on the five key objectives of ACCESS OM, namely early identification, rapid access, appropriate care, continuity of care, and youth and family engagement. A community mapping process was conducted at the beginning of the transformation to help develop a comprehensive inventory of services, identify challenges and optimize partnerships to address the five key objectives.

**Results:**

The following strategies represent key elements in the transformation: coordination and partnerships between hospital, community and voluntary organizations, as well as different sectors of the community (e.g., Child and Youth Services, Education, Community Safety and Correctional Services, CSCS); working with local champions (e.g., Youth Diversion Officer and the Mental Health and Addictions Nurse in the school sectors); establishing a youth‐friendly space in a central part of the community, where services are co‐located and operate within an open‐concept design; training of ACCESS Clinicians to conduct an initial assessment; engaging youth and family in service‐level recruitment, planning, daily operations, and evaluation, including hiring of youth and family peer navigators; and, engaging the community through awareness and educational events.

**Conclusions:**

The success of this transformation needs to be measured on various outcome parameters, but it is notable that neighbouring communities are already beginning to implement a similar model.

## 1 INTRODUCTION

Young people in Canada experience significant challenges in accessing mental health services, including delayed identification of mental health issues, long wait lists and abrupt transitions in care (Malla et al., 2018). These barriers are compounded in rural, remote, and Indigenous communities where geographic, economic and cultural factors also influence access to services (Boydell et al., 2006). To respond to these challenges, ACCESS Open Minds (ACCESS OM), a pan‐Canadian project aiming to transform and evaluate the way mental health services are delivered to youth between the ages of 11 and 25 in 14 diverse communities, was initiated in 2014 (Malla et al., 2018). In this study, we describe how service transformation occurred during the initial implementation phase in the semi‐urban and rural region of Chatham‐Kent.

## 2 COMMUNITY CONTEXT

Chatham‐Kent is mostly a rural municipality, located in the south‐western region of Ontario, Canada with a population of 101 647, including 11 595 residents between the ages of 15 and 24. The vast majority of residents speak only English (97 790) with a minority speaking only French (2245); 5270 persons indicate being of Indigenous origin (Statistics Canada, 2017). Homelessness has become an issue in this municipality and there have been recent efforts to address this, for example, through the implementation of a Housing First Homes 4 Youth (Homes for Good) Intensive Case Management Supportive Housing program (Chatham‐Kent Housing & Homelessness, 2017).

### 2.1 Status of youth mental health services prior to ACCESS Open Minds transformation

Historically, mental health services in Chatham‐Kent have been delivered through a collaborative structure between a public hospital and a community organization. This structure includes: (a) the Chatham‐Kent Health Alliance (CKHA), a publicly funded hospital providing inpatient and outpatient mental health and addiction services and (b) the Canadian Mental Health Association Lambton‐Kent (CMHA‐LK), a national not‐for‐profit organization promoting mental health and providing recovery‐oriented mental health services. An integrated leadership approach, wherein both organizations are led by the same Director of Operations and the Chief of Psychiatry, facilitates continuity of care, efficient use of human resources, and sharing of data through an electronic health record system. Within this context, six psychiatrists provide specialized mental health services (e.g., eating disorders, addictions and first episode psychosis), general psychiatric services for long‐term care, and telepsychiatry services for individuals in rural communities. Moreover, children and adolescents with mental health issues were referred to CKHA or to Chatham‐Kent Children's Services.

### 2.2 Challenges pertaining to youth mental healthcare

Despite the unique collaborative structure between the hospital (CKHA) and community organization (CMHA‐LK), youth visits to hospital emergency departments and demand for services were increasing each year. Navigating the system was complicated for youth and their families with several factors contributing to these challenges, including: (a) services operating in a siloed manner with significant overlap; for example, Chatham‐Kent Children's Services provided mental health services to individuals under 18, while the CMHA‐LK offered services to individuals 16 and over, resulting in uncertainty regarding where individuals between 16 and 18 should access care; (b) lack of coordination among mental health education and awareness initiatives; (c) limited access to a child psychiatrist with paediatricians often filling this void and referring youth to a local psychiatrist for shared care or consultation; (d) no inpatient child and youth mental health services; and (e) minimal awareness of existing protocols, frameworks or structures to coordinate operations among community organizations providing services to youth.

## 3 TRANSFORMING YOUTH MENTAL HEALTH SERVICES IN CHATHAM‐KENT

### 3.1 Community mapping

In January 2015, a community stakeholder meeting was facilitated by an international leader in innovation and change management who had been involved in many of the region's discussions related to youth mental health services. The purpose of the meeting was to introduce agencies delivering youth‐focused services to the opportunity of establishing a youth hub in Chatham‐Kent. Stakeholders attending this meeting included mental health and addictions services; social services, such as housing and employment; partners in the education sector; youth police diversion services and community organizations.

At the beginning of the meeting, each agency identified gaps in youth mental health services; however, as this process unfolded, the list of gaps reduced significantly as each agency became aware of the services that existed in the community. Next, an operational planning working group was formed to develop a comprehensive list of existing mental health and related community resources and to identify gaps in services. Two ACCESS OM ambassadors (a family peer navigator (PN) and a community volunteer) then took the lead in establishing a more detailed inventory of services and gaps. They approached each agency, inquiring about resources and services, compiling a list of stakeholders, their program type, and their type of contribution to the service transformation, as illustrated in Table 1.

**Table 1:** Description of stakeholder organizations (listed alphabetically) and their involvement in the transformed service

Agency	Program type	Frequency	Description
ACCESS Open Minds (ACCESS OM) Youth Advisory Council	Core/Peer Support	As needed	Chatham‐Kent youth who contribute to ACCESS OM by providing expertise to ensure that the ACCESS OM Chatham‐Kent site is youth friendly
Canadian Mental Health Association (CMHA) MH First Responder Case Manager	Core/Adjunct	Two permanent offices; and on an as needed “walk‐in” basis	Youth Transitional Case Management: Provides case management services with a strength‐based, goal‐oriented approach that empowers and provides clients with the tools to independent recovery First Response Team: Initial first assessment and triage into mental health and related community services
Chatham‐Kent Children's Service	Core	Daily	Provides counselling sessions (non‐crisis and crisis) and brief services (single sessions for non‐crisis situations and walk‐in clinic). Provides mental health assessments or inpatient care
Chatham‐Kent Police Services	Adjunct	As needed	Provides an alternative for youth to receive counselling and education while providing restitution to the victim instead of the youth being charged with the offence involved and attending court resulting in a possible criminal record
Chatham‐Kent Public Health Unit	Core/Adjunct	As needed	Provides smoking cessation, counselling and support (group or individual). Also offers mental health/well‐being programs (friends programming and teaching cognitive and emotional skills)
Chatham‐Kent Recreation	Adjunct	As needed; provides offsite opportunity	Provides recreation opportunities such as swimming, arenas, fit parks multi‐trails, parks and open spaces. Goal is to get “more people, more active, more often” in a positive, safe environment
Community Care Access Centre‐Erie St. Clair (CCAC)	Adjunct		Provides school support, assessments, interventions and support to students with mental health and or addictions issues. Provides consultation and education services to district school board staff in relation to mental health and/or addictions
Family Service Kent (FSK)	Adjunct	Daily	KIDS Team coordinates access to various services and supports for children/adolescents (0‐18) with complex needs that may require a response from more than one service provider
Lambton Kent District School Board (LKDSB)	Core/Section 23/Adjunct	As needed	Connecting to school mental health supports including psychoeducational clinicians, social workers, and child and youth workers
Make Children Better Now	Adjunct	As needed	Offers one‐on‐one counselling to victims, bystanders and family for the betterment of a “child's life” through active listening in a non‐judgmental and empathetic way
Mental Health and Addictions Program (MHAP)	Core	Daily	Provides early intervention, assessment and treatment for individuals experiencing early signs and symptoms of psychosis. In addition, provides Mental Health and Addictions counselling services inclusive of eating disorders therapy
Mental Health Network (MHN)	Core	As needed	Peer support and daily health and wellness programming for individuals with mental illness
Rain & Shine Behavioural Counselling	Core/Adjunct	As needed	Counselling and programs utilizing Applied Behavioural Analysis and Cognitive Behavioural Therapy to address behavioural issues
St. Clair Catholic School Board (SCCSB)	Adjunct	As needed	Connecting to school mental health supports including psychoeducational clinicians, social workers, and child and youth workers
Restorative Justice	Adjunct	As needed	Alternative pathways for youth 7‐17 who are at risk for delinquent, negative behaviours, and may be struggling in school, at home, and/or within their social environments
Today Not Tomorrow (TNT)—Parent Group (Early Intervention for Psychosis)	Core/Adjunct/Peer Support	Daily/as needed	Provides recovery‐based program offering support, information and tools to families whose loved ones have had a first‐time psychosis
Western Area Youth Services (WAYS)	Core/Adjunct	As needed	Goal‐focused counselling and mental health support, including telephone crisis support

## 4 MEETING ACCESS OM OBJECTIVES

Building on the insights gained through community mapping, ACCESS OM Chatham‐Kent created capacity by bringing together existing resources to ensure that youth in need were connected to the appropriate providers. The aim was to reduce duplication of resources and address the disjointed experience of youth and their families in going from agency to agency, sometimes receiving overlapping or even contradictory services. Various youth services were integrated to optimize continuity of care and increase capacity through efficient sharing of resources and responsibilities. Next, we describe the specific strategies used in addressing the five primary objectives of the ACCESS OM model of service transformation.

### 4.1 Early identification

ACCESS OM Chatham‐Kent has implemented several strategies to improve early identification of youth in need of services. For example, the site has conducted community‐wide education to increase mental health awareness and promote the service as a central point for youth referrals, targeting stakeholders at the frontline and leadership level including: two Chatham‐Kent School Boards, Home and Community Care, Mental Health and Addiction Nurses, Chatham‐Kent Police Services Youth Officer, Primary and Specialized Practitioners, the CKHA Mental Health and Addictions Program, and the CMHA‐LK. A mental health promotion specialist, employed by CMHA‐LK, has delivered presentations at work, school, and public education events on early identification, basic coping strategies, information on where youth could seek mental health services, and what to expect from ACCESS OM Chatham‐Kent. This specialist also collaborated with the Youth Diversion Officer in giving presentations to service clubs including Cogeco programs, May Court Club, the Lions Club and the Rotary Club.

All community organizations providing youth‐focused services were invited to the site's initial launch in May 2016 and the grand opening of its permanent physical space in May 2017. Organizations set up booths in the new physical space to promote their work, which also provided an opportunity for strengthening mutual awareness and inter‐organizational connections. In addition, ACCESS OM Chatham‐Kent, through a partnership agreement, engaged with the Municipality of Chatham‐Kent's income and housing program (“Homes 4 Youth—Home for Good”) to identify youth at risk of becoming homeless (e.g., those who have dropped out of high school) and provide them with safe and affordable housing.

**Figure 1:**
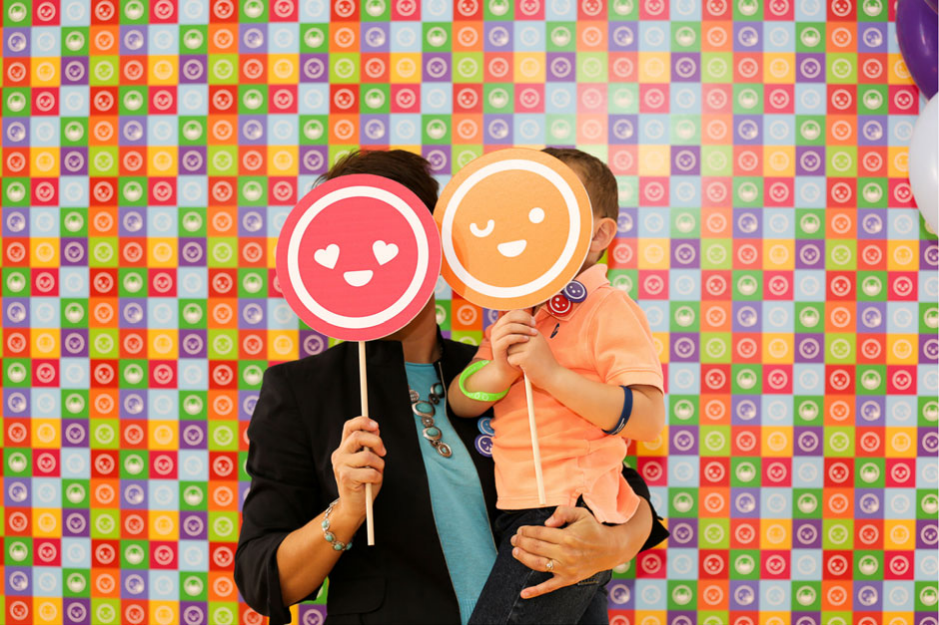
Illustration of “What's your emoji?” marketing materials, co‐designed by young people, used at community events

As part of the community education initiatives, youth have been involved in creating visually appealing marketing materials (e.g., pins, posters, business cards) using the slogan “What's your emoji?” (see Figure 1). These materials are disseminated during community awareness events with the aim of engaging audiences in a fun and interactive way in discussing how to recognize, relate to, and help youth connect to mental healthcare. ACCESS OM Chatham‐Kent has also participated in radio interviews, has been featured in local news articles, and has disseminated agency‐specific communications for broader community exposure. In addition, the site manages a website and a Twitter account, and it has hosted a well‐publicized Twitter live‐chat targeting youth.

**Figure 2:**
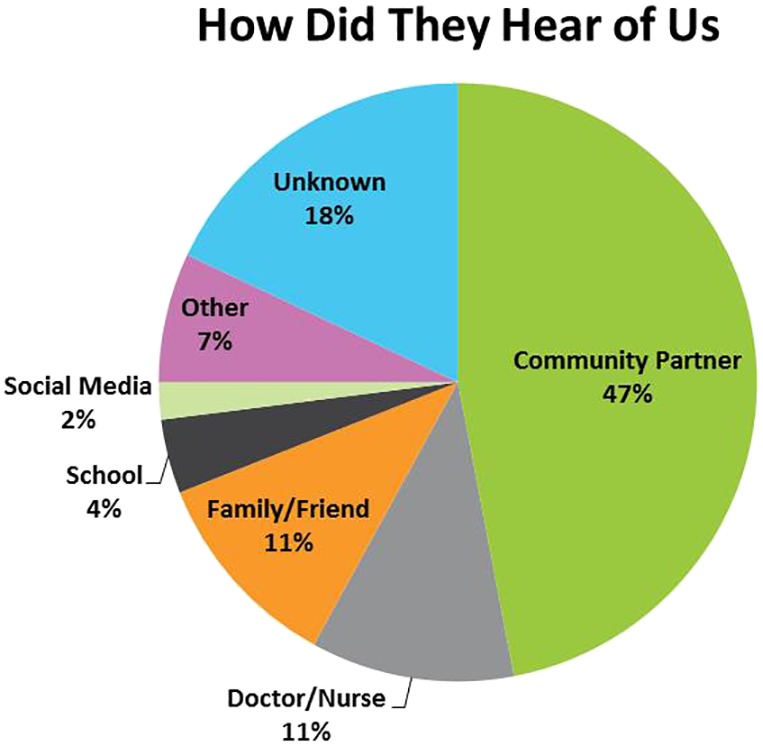
Summary of how youth learned about ACCESS Open Minds Chatham‐Kent

In the first year of ACCESS OM Chatham‐Kent operation (May 2016‐March 2017) there was an increase in the numbers of youth seeking mental healthcare by approximately 25% in comparison to the previous 12 months, including adolescents aged 15 and under. This increase could be attributed to the prominent location of the new physical space (ie, downtown centre), coordinated approach between partnering organizations, and awareness activities. Figure 2 illustrates how youth have learned about ACCESS OM Chatham‐Kent and the key role community partners have had in this process.

### 4.2 Rapid access

To facilitate rapid access to an assessment within 72 hours (an ACCESS OM objective), a “youth space” was developed as the “go‐to” place for youth, families, and friends seeking mental health‐related support and services. Many core services (e.g., counselling, housing, case management and psychiatry) have been co‐located in this youth space using an open‐concept design. This co‐location has removed barriers to care through removing walls, transforming the way service providers conduct their daily work. This approach prevents individuals from being unnecessarily referred out to multiple locations and aims to improve continuity of care through a coordinated, unified approach to treatment planning.

Youth and family peer workers are the initial points of contact helping to engage individuals seeking help and identifying their needs. Including peer workers at the initial point of care aims to reduce the demand for specialized services (e.g., psychiatric consultation) and avoid unnecessary services as in our experience, sometimes all that is needed is to be heard and supported from someone with live experience. Youth have access to an inter‐professional team, including ACCESS Clinicians (social workers, nurses and occupational therapist), trained to conduct an informative and engaging initial evaluation. A clinical coordinator/social worker assists with triaging individuals who need case management support vs counselling vs psychiatric consultations. When an initial evaluation indicates that a youth may be in acute crisis, a mental health nurse from the CKHA is invited to the youth space, and accompanies the youth to emergency or inpatient services, whenever possible. This procedure aims to facilitate a less traumatic experience for youth who may need inpatient care. Another key strategy deployed to facilitate rapid access is that no referral or clinical diagnosis is required to receive ACCESS OM services.

### 4.3 Appropriate care in 30 days

The majority of services provided by the site are located within shared office space, facilitating access to appropriate care within 30 days. These services include: (a) youth and family support, including a dialectical behaviour therapy skill‐based group for family members called “Family Connections” providing education and skills (e.g., emotion regulation, problem management, effective communication) to effectively engage with youth; (b) psychological therapy for up to 14 sessions as per provincial guidelines; (c) Housing First Homes 4 Youth program; (d) single‐session walk‐in therapy two days a week for individuals who are not in crisis, not judged to have serious mental health problems such as psychosis, bipolar disorder or major depression, and are seeking an immediate response. Single‐session therapy with a professional counsellor is based on the ideas that most clients can benefit from a single session; in many cases one session may be all that individuals attend; and for many, a single session is sufficient to reduce distress (Hymmen, Stalker, & Cait, 2013; Slive, McElheran, & Lawson, 2008; Stalker et al., 2016). Single‐session therapy is a point of entry into care, providing an opportunity to engage youth, offer immediate services, and proceed to an ACCESS OM assessment. This immediate access is designed to make services available to individuals who are ready to access therapy and reduce the issue of no‐shows; (e) consultation and treatment provided by .3 FTE (1.5 days) psychiatrists; (f) life skills training and focused specialized services, including the Today Not Tomorrow early intervention for psychosis service and (g) social networking groups.

Youth requiring interventions not offered in the ACCESS OM space (e.g., long‐term follow‐up for bipolar disorder) are referred to appropriate services, with support provided to youth and families until the appropriate intervention commences. Such referrals and navigation support are facilitated by strong links between ACCESS OM and other mental health services in the community, and staff from these other services who spend time in the ACCESS OM space.

### 4.4 Continuity of care

ACCESS OM Chatham‐Kent was built on pre‐existing work towards integration to improve youth experiences of mental health services. This preliminary effort has resulted in a fundamental partnership between children's mental health services, hospital, and the adult mental health sector, and has produced strategies for improving continuity of care. For example, the emergency department diversion program was established two years prior to ACCESS OM, where children's services were on‐call to complete an assessment for any youth arriving in the emergency department. This practice is helping to raise awareness in terms of what community services can offer as an alternative to hospital admission. Furthermore, with Chatham‐Kent Children's Services offering services within the ACCESS OM physical space, youth no longer experience the abrupt change at age 18 in terms of where they receive services. Youth can continue to receive services in the same location and maintain proximity to a former counsellor. Counsellors facilitate seamless transitions through introducing and accompanying youth in their first interactions with new counsellors, and even continuing to provide services using a shared care approach, while the client establishes a therapeutic relationship with the new counsellor. Youth and family have expressed appreciation for being able to receive services in a familiar setting, including being greeting by the same receptionist who has received them in the past.

### 4.5 Youth and family engagement

Youth and families have been actively involved in the establishment and operation of the site, raising awareness in the community, and providing leadership on youth and family engagement at the national level of the network.

*Establishing the site and contributing to its daily operations*. Members of the site's youth advisory committee have contributed to choosing the location of the space, meeting with realtors, touring potential facilities, and designing the space. The latter included selecting themes for individual rooms and contributing ideas on the “look and feel” of the common space, which has been decorated with youth‐made art. This process contributed to youths' decision‐making skills, as they often needed to make choices in relation to feasibility and priorities.

Youth and families participate in the site's daily operations. The program employs two Youth PNs and a Family Navigator; they are often the first individuals whom visitors meet upon entering the centre and are part of the clinical team. They are trained in Mental Health First Aid (Kitchener & Jorm, 2002) and active listening, and aim to provide empathic support, helping visitors feel like they are not alone. This can also be a fulfilling employment opportunity for those who have experienced the mental healthcare system and subsequently want to help their peers. Youth and families also contribute to decisions regarding staff hiring by participating in interviews and sharing feedback on candidates' ability to engage, level of passion, shared vision, and understanding of ACCESS OM. In terms of program planning and evaluation, the Family Navigator participates in the Quality Committee, challenging existing practices and providing feedback on new initiatives, and the Youth Navigators are trained to support data collection activities for research and evaluation.

*Promoting ACCESS OM*. Youth and family representatives have become site ambassadors, attending all promotional events, participating in radio interviews, hosting the launch events and giving public presentations. Some of the youth and family representatives have employed positions within the site (e.g., PNs) whereas others are volunteers who are often compensated through gift cards, meals and reimbursement for travel. Leaders in the community, healthcare and social services, private funders, and policy‐makers have been eager to hear directly from youth, seeking their approval on every issue and event.

*Providing leadership and support at the national level*. Youth and family leaders from the site are members of the ACCESS OM National Youth and Family and Carers Councils and have served as role models to other ACCESS OM sites in youth and family engagement practices (e.g., supporting ACCESS OM Edmonton in their creation of a family peer support position to offer Family Connections groups). This modelling role has further empowered Chatham‐Kent's youth and family leaders.

## 5 ACTIVE INVOLVEMENT OF VOLUNTARY ORGANIZATIONS AND COMMUNITY MEMBERS

Community engagement has been instrumental to the establishment of the physical space and the services offered. For example, a local contractor who contributed to the street‐scape design of the space volunteered services for additional projects, stating, “If this service existed when I was younger, I would have looked for help.” Moreover, the Chatham‐Kent community has supported ACCESS OM in various ways, including unsolicited donations from local high schools, philanthropic organizations, private industry and family members. The Rotary Club has played a consistent cornerstone role by providing seed funding for programs, and donations for furnishings and numerous public events.

## 6 RESEARCH AND EVALUATION

Service evaluation is conducted through the implementation of the ACCESS OM Evaluation Protocol. To facilitate engaging youth in both clinical and research activities, we created a joint position integrating PN and Research Assistant (RA) functions. The PN/RAs received training from the ACCESS OM central office and contribute to ongoing data collection at the site. Having two PN/RAs share roles ensures that they do not duplicate work and are able to support each other. Integrating peer navigation with research/evaluation aims to ensure continuity, team communication and youth understanding of the importance of research/evaluation in youth mental healthcare.

## 7 SUSTAINABILITY

Several strategies are being implemented towards achieving sustainability. For example, participating organizations are identifying opportunities to maximize the use of existing resources and funds that have been invested in the ACCESS OM model. To illustrate, the early psychosis program has expanded its scope to providing services for individuals experiencing anxiety and mood disorders. In addition, the site has participated in numerous local, regional, provincial, and national presentations to further promote ACCESS OM as a core component of the community. Community champions such as the Youth Diversion Officer and the Mental Health and Addictions Nurse in the school sectors continue to introduce ACCESS OM as a key resource for youth seeking help. Although the donations received from the community are one‐time gifts, and sources of sustainable base funding continue to be explored, donors have expressed the value that ACCESS OM brings to the community and recognize the critical need for it to remain a core service.

Inter‐ministerial and private sector collaborations have also contributed to sustainability of ACCESS OM Chatham‐Kent. Involvement of other provincial ministries, including Health, Child and Youth Services, Education, Police Services and the local municipality, are indicators of the community coming together to transform youth mental health services. Furthermore, the Ontario Ministry of Health is now committing to an initial investment of 10 hubs to promote youth wellness in Ontario (Youth Wellness Hubs Ontario, YWHO), one of them being in Chatham‐Kent.

## 8 COMMUNITY IMPACT

The site team was the proud host for the launch of the provincial YWHO initiative, where leaders, front‐line staff, youth, and peers from outside the community visited the site to witness what can be achieved in a modest‐sized community. The site team has also been overwhelmed with meeting requests, site tours, and formal conference presentations, and has taken time to accommodate each one, recognizing the importance of building awareness about such an innovative youth mental health endeavour. There are current efforts to expand similar services to a neighbouring city (ie, Sarnia, Ontario); this new project is already garnering similar attention and coalescing local passion as in Chatham‐Kent.

## 9 DISCUSSION

We have described how the five objectives of ACCESS OM have been implemented in a mostly rural and semi‐urban Canadian community to improve mental health services for youth. A key element of the transformation has been to establish a youth‐friendly space in a central part of the community, where services are co‐located and operate within an open‐concept design. Within this shared‐space, youth and family engagement is integral to the planning, operations, evaluation and promotion of services. ACCESS Clinicians are critical to ensuring youth receive informative and engaging initial assessments and care. Coordination and partnerships between hospital, community and voluntary organizations; engagement of different service sectors (e.g., Child and Youth Services, Education, Police Services); and working with local champions (e.g., Youth Diversion Officer), have been core strategies for facilitating early identification of youth in need and supporting sustainability.

## 10 CHALLENGES

Several challenges have been experienced in transforming the services; these pertain to shared‐decision‐making between stakeholders, recruitment and retention of qualified staff in a semi‐urban and rural setting, staff going on temporary leave, acquiring sustainable funding, providing services to individuals living in rural settings (including Indigenous communities) located at a distance from the ACCESS OM Chatham‐Kent physical site, and maintaining capacity to deliver services with increasing numbers of youth being referred. To address these challenges, ACCESS OM Chatham‐Kent has made progress towards improving access to remote communities through embracing technology and creating community partnerships in various rural locations in an effort to improve awareness, early detection of mental illness, and strengthen linkages with AOM services. The next stage of transformation will be focussed on developing and implementing strategies to address the needs of youth living in Indigenous communities.

Working in a model of shared‐decision‐making between stakeholder groups has also been a learning process. For example, during the renovations of the youth space, contractors created a graffiti wall without the knowledge of the youth advisory committee. This initially invoked a sense of panic among the leadership team since the decision had been made without youth involvement. Although it was an uncomfortable unveiling, the youth advisory council was pleasantly surprised. This incident reinforced the value of transparency and the practice of “nothing for youth, without youth.”

Establishing a human resource base with a clear understanding of ACCESS OM's objectives has also been a key challenge. There is a need for therapists, community social workers, and psychiatrists who can engage youth and appreciate the challenges that transitional age youth experience. Sustainable funding is also a challenge. Grant proposals and business case submissions have helped to maintain funding for ACCESS OM. The Chatham‐Kent site is optimistic that this model is now on the radar of their provincial Ministry of Health, as evidenced through the investment towards the YWHO initiative. While helpful in building awareness and promoting buy‐in for sustainability, engagement and education events take resources away from direct services. This responsibility has now mostly shifted to a mental health promotion specialist, thus reducing demand on direct service providers.

## 11 CONCLUSION

ACCESS OM Chatham‐Kent has the privilege of being the only site in Ontario to participate in the pan‐Canadian ACCESS OM project. A sense of commitment among various community stakeholders has been instrumental in driving youth mental health services transformation in Chatham‐Kent. The transformation has entailed partnership between mental health and addiction services, youth and families, community organizations and volunteer organizations. The lessons learned from establishing this youth‐oriented model serve as inspiration for similar projects, particularly in similar geographic and organizational contexts.
